# Prenatal exposure to opioid maintenance treatment and neonatal outcomes: Nationwide registry studies from the Czech Republic and Norway

**DOI:** 10.1002/prp2.501

**Published:** 2019-08-14

**Authors:** Marte Handal, Blanka Nechanská, Svetlana Skurtveit, Ingunn Olea Lund, Roman Gabrhelík, Anders Engeland, Viktor Mravčík

**Affiliations:** ^1^ Department of Mental Disorders Norwegian Institute of Public Health Oslo Norway; ^2^ Department of Addictology, First Faculty of Medicine Charles University Prague Czech Republic; ^3^ Institute of Health Information and Statistics of the Czech Republic Prague Czech Republic; ^4^ Norwegian Centre for Addiction Research at the University of Oslo Oslo Norway; ^5^ Department of Chronic Diseases and Ageing Norwegian Institute of Public Health Oslo Norway; ^6^ Department of Global Public Health and Primary Care University of Bergen Bergen Norway; ^7^ National Monitoring Centre for Drugs and Addiction Office of the Government of the Czech Republic Prague Czech Republic

**Keywords:** buprenorphine, methadone, neonate, opioid maintenance treatment, prenatal exposure

## Abstract

There is lack of knowledge about the safety of treatment with methadone and buprenorphine as part of opioid maintenance treatment (OMT) during pregnancy. The purpose of this study was to examine neonatal outcomes concerning the use of OMT during pregnancy. We used nationwide registry linkages from the Czech Republic (2000‐2014) and Norway (2004‐2013). We compared prenatally OMT–exposed newborns with (a) newborns of women hospitalized with opioid use disorder during pregnancy in the Czech sample and (b) newborns with neonatal abstinence syndrome (NAS) in Norway. We performed multivariate linear and binary logistic regression exploring the associations between OMT and neonatal outcomes (growth parameters, gestational age, fetal death, small for gestational age, Apgar score, and NAS). Regression coefficients (*b*) and odds ratios (ORs) were estimated. The cohorts consisted of 333 Czech, and 235 Norwegian OMT–exposed newborns, and 106 and 294 newborns in the comparison groups, respectively. In both countries, the neonatal growth parameters were similar in the OMT and the comparison groups. In Norway, OMT exposure prolonged gestational age (adjusted *b* = 0.96 weeks, 95% confidence interval [CI] =0.39‐1.53) while the odds of preterm birth and Apgar score at 5 minutes were lower than in the comparison group (adjusted OR = 0.35, 0.16‐0.75 and aOR = 0.21, 0.06‐0.78, respectively). Newborns of women in OMT had similar growth parameters as newborns of women with opioid use disorders who were not in OMT during pregnancy. Overall, our findings do not suggest that OMT results in worse neonatal outcomes.

**Abbreviations**
ATC: Anatomical Therapeutic Chemical
NAS: neonatal abstinence syndrome
OMT: Opioid maintenance treatment
ORs: odds ratios
SGA: small for gestational age

## 1 INTRODUCTION AND BACKGROUND

Opioid use disorder is characterized by the persistent use of opioids despite the adverse consequences of its use. Opioid maintenance treatment (OMT) can stabilize women and reduce the risk of relapse to illicit opioid use.^1^ The World Health Organization (WHO) strongly recommends women with opioid use disorders to continue or commence OMT with either methadone or buprenorphine if they become pregnant, despite the meager evidence behind this recommendation.^2^

Previous research has emphasized benefits with OMT during pregnancy. If left untreated, opioid use disorders during pregnancy are associated with a range of obstetric and neonatal complications, such as spontaneous abortions, intrauterine growth retardation, preterm birth, and low Apgar score.^3^ Since methadone maintenance treatment was introduced in the US in the late 1960s, studies have reported beneficial effects, such as reduced incidence of obstetric and fetal complications, neonatal morbidity and mortality, from methadone therapy compared to illicit heroin use during pregnancy.^4^, ^5^, ^6^ Maintenance treatment with buprenorphine has later been shown to be comparable to that of methadone.^7^, ^8^

OMT is, however, also associated with disadvantages. Neonates exposed to opioid agonist therapy have higher rates of adverse neonatal outcomes compared to neonates in the general population. There is evidence of high rates of neonatal abstinence syndrome (NAS), lower birth weight, length, and head circumference as well as increased rates of perinatal mortality.^9^, ^10^, ^11^ The incidence and severity of NAS are not only higher after opioid agonist therapy when compared to the general population; it seems to be higher after exposure to methadone than after exposure to heroin.^6^

With an increasing focus on preventing harm to the unborn child, the low evidence behind OMT has resulted in critical questions from pregnant woman, clinicians, researchers,^12^, ^13^ policymakers, and politicians about the safety of OMT during pregnancy.

Previous studies have several weaknesses, including one major concern of unmeasured confounding. Most studies only compare newborns of women in OMT during pregnancy with newborns of women from the general population. Women in these two groups have very different confounder distributions, and comparison between them is therefore not suitable. Using appropriate comparison groups is one way to come closer to an unbiased association. Many of the studies were also conducted several years ago, and often in selected populations. Many were performed in countries where the quality of health care differs for different patient populations, making a comparison between groups difficult.^7^, ^14^, ^15^, ^16^, ^17^, ^18^

Moreover, OMT has changed substantially since methadone was first introduced and since the early studies were conducted: more pharmacological options have been introduced, and inclusion and exclusion criteria for OMT have changed. In light of these changes, exploring these questions in new settings and using new approaches may contribute to shed more light on the problem. Use of data from nationwide health registries is a new approach that makes it possible to address several of the limitations from earlier studies.^19^ Linking data from multiple registries allows for large, unselected study populations and identification of relevant comparison groups.

To increase the knowledge about the safety of OMT treatment during pregnancy, we utilize nationwide registry data from two different European countries to study associations between OMT exposure and adverse neonatal outcomes in newborns. To reduce the problem of unmeasured confounding, we compared newborns born to women who were in OMT during pregnancy to newborns whose mothers had indications of opioid use disorders but who were not in OMT during pregnancy.

## 2 MATERIALS AND METHODS

### 2.1 Study design

The study is an observational cohort study with two national cohorts based on linkages of nationwide registries.

### 2.2 Setting

OMT became available for pregnant women in 1997 in the Czech Republic and in 1998 in Norway.^20^, ^21^ In both countries, OMT is provided predominantly in an outpatient setting where methadone, buprenorphine, and buprenorphine/naloxone are used as drugs. In the Czech Republic, methadone is dispensed in specialized facilities free of charge, while buprenorphine–based drugs are available in community pharmacies and are typically fully paid by the patient. In Norway, most pregnant women in OMT receive their OMT drugs at pharmacies free of charge as part of a national OMT program.^22^

### 2.3 Data sources

Both the Czech Republic and the Norway have nationwide registries with personal identification numbers. This enables linkages of data between different registries on an individual level, and on family levels such as between mother and child. A more detailed description of the data sources is provided elsewhere.^8^, ^22^, ^23^

### 2.4 Registries in the Czech Republic

In the Czech Republic, physicians are obliged by law to report data to the national health registries.

#### 2.4.1 National Registry of Reproduction Health (NRRH)

The NRRH includes information about all women and their children during pregnancy, delivery, and puerperium. For instance, maternal variables such as demographic and socioeconomic data, alcohol, tobacco, and illegal drug use during pregnancy and information about the delivery are included in the registry. Information about the newborn, such as birth parameters, congenital malformations, and death, is also included.

#### 2.4.2 The National Registry of Addiction Treatment (NRAT)

The NRAT contains data on all patients starting and terminating different addiction treatments. It includes, for instance, information about patients who receive OMT, such as date of treatment initiation and termination and type of OMT drug.

#### 2.4.3 The National Registry of Hospitalized Patients

The NRIT includes information on every episode of all types of hospitalizations, including information on dates of admission and discharge from the hospital. Diagnoses on the discharge summary are coded according to the International Statistical Classification of Diseases, 10th Revision (ICD‐10).

### 2.5 Registries in Norway

#### 2.5.1 The Medical Birth Registry of Norway (MBRN)

The MBRN is based on a compulsory notification of every birth or late abortion from physicians or midwives attending the birth. The MBRN includes information concerning all births and late abortions from the 12th gestational week and onwards. The registry includes information concerning pregnancy, delivery, and the newborn. Maternal data are also collected, such as demographic and socioeconomic backgrounds of the pregnant women and also tobacco smoking during pregnancy. Information about neonatal outcomes, such as gestational age, birth parameters, NAS, and congenital malformations, are also included.

#### 2.5.2 The Norwegian Prescription Database (NorPD)

Pharmacies are obliged by law to forward prescription data to the NorPD. The NorPD includes information about all prescription drugs, including OMT drugs, dispensed at pharmacies to patients in ambulatory care. For each prescription, the dispensing date and detailed information on the drug is registered. The drugs are classified according to the Anatomical Therapeutic Chemical (ATC) classification system.^24^

#### 2.5.3 Statistics Norway (SSB)

From Statistics Norway we included information about maternal education. Educational institutions are obliged to report completed education on an individual level to SSB.

### 2.6 Study population and study period

The study populations were pregnant women and their children born during the study period: 2000‐2014 in the Czech Republic (N = 1 547 273) and 2004‐2013 in Norway (N = 554 310). From this population, we identified women who had indications of opioid use disorders during pregnancy, who were either in OMT during pregnancy or not in OMT.

### 2.7 Exposure to OMT drugs during pregnancy

Exposure was defined as the use of the OMT drugs methadone or buprenorphine during pregnancy. In the Czech Republic, data on initiation and termination of drug treatment from the NRRH were used to identify if women were receiving OMT drugs during pregnancy. Of the 333 women who were in OMT treatment during pregnancy, 192 (58%) were in OMT throughout the entire pregnancy, while 141 (42%) were in OMT for only parts of pregnancy (mean 115 days of treatment, minimum 6 days and maximum 250 days).

In Norway, NorPD was used to identify women using OMT drugs. Those who were dispensed OMT drugs at least once during pregnancy were defined as exposed to OMT during pregnancy. More than 95% of all OMT women receive more than one prescription of an OMT drug during pregnancy. Pregnant women in OMT receive, on average, a total amount of buprenorphine corresponding to approximately 10 mg/day in pregnancy, while women who use methadone on average receive a total amount of about 65 mg/day. Approximately 80% of the OMT drugs are dispensed at pharmacies both early and late in the pregnancy. This suggests that they used these drugs throughout their pregnancy.

### 2.8 Outcomes

The outcomes studied were neonatal outcomes identified in the NRRH or MBRN, and included: gestational age, preterm birth (<37 weeks of gestation), growth parameters (birth weight, length and head circumference), small for gestational age (SGA),^25^ miscarriage (death of a fetus between gestational week 12 and 22), stillbirth (death of a fetus in gestational week 22 or later), NAS (only in the Norwegian cohort), and Apgar scores < 7 at 1 and 5 minutes.

### 2.9 Confounding variables

We obtained information on sociodemographic variables and lifestyle from the NRRH in the Czech Republic and from the MBRN and SSB in Norway. This information included age and marital status (registered as not married, married or unknown; in Norway, the married category also included living with a partner). Information about education was provided in the following categories: primary, secondary, university, or unknown. Information about occupation was only available in the Czech Republic, and included the categories unemployed, employed and unknown. Information on recreational drug use during pregnancy, that is, use of alcohol and illicit drugs was only available in the Czech Republic. Information about tobacco smoking was available in both countries and categorized as yes, no, or unknown.

### 2.10 Comparison groups

To be able to study the safety of the OMT in pregnancy we created relevant but different comparison groups in the two countries by identifying pregnant women with indications of opioid use disorders who were not in OMT during pregnancy.

For the Czech comparison group, we selected pregnant women who, according to the NRIT, had been hospitalized during pregnancy with a diagnosis at discharge indicating that she had an opioid use disorder (F11.X “mental or behavioral disorder due to opioid use” according to the International Statistical Classification of Diseases, 10th Revision [ICD‐10]). Furthermore, the women could not have received OMT during pregnancy, according to the NRAT.

The Norwegian control group comprised of women who, according to data in the MBRN, gave birth to a child with NAS. Newborns with NAS were recognized if they have received an ICD‐10 diagnosis of “neonatal withdrawal symptoms from maternal use of drugs of addiction” (P96.1 according to ICD‐10). When reporting to the registry, the midwife or physician could also tick a box for abstinence if neonatal irritability and neurological symptoms had been observed and there was documentation of maternal abuse of prescription drugs, alcohol or illicit drugs during pregnancy, or the woman herself gave such information. Furthermore, the women in the comparison group could not have received OMT during pregnancy, according to the NorPD.

### 2.11 Analysis strategy and statistics

In a previous study using the same nationwide registry data as in this study,^8^ we did not find any significant differences in risks of adverse neonatal outcomes between prenatal exposure to methadone or buprenorphine. Thus in this study, we collapsed the buprenorphine and methadone–treated women into one OMT–exposed group in each country. Then, in each country, we compared the OMT group with the comparison group in that country. First, we present sociodemographic background and substance use during pregnancy. Next, we focus on descriptive statistics (mean, standard deviation, percentages with 95% confidence intervals) on neonatal outcomes, restricted to singleton births in both countries. Growth parameters (except SGA) were restricted to term births (≥37 gestational weeks). Gestational age, SGA, and Apgar scores were restricted to live births. Confidence Intervals for proportions were calculated using the continuity–corrected score interval method.^26^ Linear and logistic regression analyses were performed to investigate the association between OMT use in pregnancy (yes/no ‐ independent variable) and different neonatal outcomes (growth parameters, gestational age, fetal death, small for gestational age, Apgar score, and NAS ‐ dependent variable). Associations were shown as regression coefficients (b) for continuous outcomes and odds ratios (ORs) for dichotomous outcomes both with 95% confidence intervals. Unadjusted and adjusted b and ORs were estimated. The following factors were included in the adjusted multivariate regression analysis: age, marital status, education, and smoking. The statistical significance level was set to 0.05. Statistical analyses were conducted using SPSS v21 for Windows.

In supplementary tables, we show maternal socioeconomic characteristics, as well as neonatal outcomes in the OMT–exposed newborns compared to newborns of women in OMT before and after (but not during) pregnancy and compared to all the children in the general population.

### 2.12 Ethics

The study was approved by the Institutional Review Board of the General University Hospital in Prague (IRB00002705), the Regional Committees for Medical and Health Research Ethics (REK, AE: 2012‐222), and the Norwegian Data Inspectorate.

## 3 RESULTS

### 3.1 Background characteristics

In the Czech Republic, 333 women were in OMT during pregnancy, while 106 had opioid use disorder without being in OMT during pregnancy. In Norway, we identified 235 women who were in OMT during pregnancy, while 294 had indications of opioid use disorder but were not in OMT during pregnancy. Tables 1 and 2 display maternal background characteristics. In both countries, women in the comparison groups were younger than women in OMT. In the Norwegian sample, the pregnant women in the OMT group had lower education levels (primary education 71.5% versus 50.0%) and higher smoking prevalence (68.5% versus 57.2%) during pregnancy than the comparison group (Table 2).

**Table 1:** Socioeconomic characteristics of Czech women with indications of opioid dependence who received opioid maintenance treatment (OMT) or not during pregnancy (2000‐2014)

	Czech Republic					
	OMT (n = 333)			No OMT^a^ (n = 106)		
	n	%	95% CI	n	%	95% CI
Age, y						
≤24	98	29.4	24.7‐34.7	74	69.8	60.0‐78.2
25‐29	139	41.7	36.4‐47.3	21	19.8	13.0‐28.9
30‐34	77	23.1	18.8‐28.1	8	7.5	3.6‐14.8
≥35	19	5.7	3.6‐8.9	3	2.8	0.7‐8.7
Marital status						
Not married	266	79.9	75.1‐84.0	87	82.1	73.2‐88.6
Married	49	14.7	11.2‐19.9	10	9.4	4.9‐17.1
Unknown	18	5.4	3.3‐8.6	9	8.5	4.2‐15.9
Education						
Primary	159	47.7	42.3‐53.3	61	57.5	47.6‐67.0
Secondary	154	46.2	40.8‐51.8	42	39.6	30.4‐49.6
University	4	1.2	0.4‐3.3	0	0	0.0‐3.4
Unknown	16	4.8	2.9‐7.8	3	2.8	0.7‐8.7
Occupation						
Unemployed	274	82.3	77.7‐86.1	96	90.6	82.3‐95.1
Employed	25	7.5	5.0‐11.0	10	9.4	4.9‐17.1
Unknown	34	10.2	7.3‐14.1	0	0	0.0‐3.4
Using of addictive substances during pregnancy						
Alcohol	17	5.1	3.1‐8.2	6	5.7	2.3‐12.4
Smoking	136	40.8	35.6‐46.4	43	40.6	31.3‐50.6
Illicit drugs	129	38.7	33.5‐44.2	43	40.6	31.3‐50.6
Deliveries by multiplicity						
Single	324	97.3	94.8‐98.7	106	100	96.6‐100.0
Twins and more	9	2.7	1.3‐5.3	0	0	0.0‐3.4

Abbreviation: CI, confidence interval

a Women hospitalized with an ICD‐10 F11 diagnosis as a primary or secondary diagnosis during pregnancy were included.

**Table 2:** Socioeconomic characteristics of Norwegian women with indications of opioid dependence who received opioid maintenance treatment (OMT) or not during pregnancy (2004‐2013)

	Norway					
	OMT (n = 235)			No OMT^a^ (n = 294)		
	n	%	95% CI	n	%	95% CI
Age, y						
≤24	18	7.7	4.7‐12.0	64	21.8	17.3‐27.0
25‐29	70	29.8	24.1‐36.1	84	28.6	23.6‐34.2
30‐34	89	37.9	31.7‐44.4	80	27.2	22.3‐32.8
≥35	58	24.7	19.4‐30.8	66	22.4	17.9‐27.7
Marital status						
Not married	91	38.7	32.5‐45.3	115	39.1	33.5‐45.0
Married/living with partner	142	60.4	53.8‐66.7	175	59.5	53.7‐65.1
Unknown	<4			4	1.4	0.4‐3.7
Education						
Primary	168	71.5	65.2‐77.1	147	50.0	44.2‐55.9
Secondary	59	25.1	19.8‐31.3	78	26.5	21.7‐32.0
University	5	2.1	0.8‐5.2	48	16.3	12.4‐21.2
Unknown	<4			21	7.1	4.6‐10.9
Smoking during pregnancy						
Yes	161	68.5	62.1‐74.3	168	57.1	51.3‐62.8
No	27	11.5	7.8‐16.4	62	21.1	16.7‐26.3
Unknown	47	20.0	15.2‐25.8	64	21.8	17.3‐27.0
Deliveries by multiplicity						
Single	229	97.4	94.3‐99.0	287	97.6	94.9‐99.0
Twins and more	6	2.6	1.0‐5.7	7	2.4	1.0‐5.1

<4 denotes less than four individuals in the group, exact numbers are not shown because of regulation from the Registries

CI, confidence interval.

a Women who gave birth to a child with neonatal abstinence syndrome

### 3.2 Neonatal outcomes – descriptive statistics

Table 3 shows that most neonatal outcomes were similar in the OMT group and the comparison group in both countries. In the Czech Republic, the gestational age was similar between the OMT‐exposed and the comparison group (38.4 weeks), while in Norway the OMT‐exposed had 0.9 weeks longer gestational age than those not exposed. Similarly the proportion with preterm birth was about the same in the OMT‐exposed and not exposed in the Czech Republic (16.9% and 14.4%), while in Norway the proportion in the OMT‐exposed was about half of that in the not exposed group (7.0% versus 15.0%). Overall, the differences in neonatal outcomes between the OMT‐exposed and not exposed were negligible in the Czech Republic. Independent of exposure to OMT or not, the mean growth parameters (birth weight, length, and head circumference) were lower in the Czech Republic than in Norway.

**Table 3:** Birth outcomes in newborns of women with indications of opioid dependence who either were in opioid maintenance treatment (OMT) or not during pregnancy in the Czech Republic (2000‐2014) and Norway (2004‐2013). Singleton pregnancy only

	Czech Republic				Norway			
	OMT (n = 324)		No OMT^b^ (n = 106)		OMT (n = 229)		No OMT^c^ (n = 287)	
	Mean	SD	Mean	SD	Mean	SD	Mean	SD
Gestational age^d^ (wk)	38.4	2.6	38.4	2.6	39.1	2.1	38.2	3.0
Birth weight^e^ (g)	3056	469	3081	404	3304	507	3286	507
Birth length^e^ (cm)	48.3	2.4	48.3	2.4	49.0	2.5	49.0	2.2
Head circumference^e^ (cm)	33.8	1.7	33.5	1.5	34.6	1.5	34.7	1.4

	n	% (95% CI)	n	% (95% CI)	n	% (95% CI)	n	% (95% CI)
Abortion induced								
Yes	—	—	—	—	<4	—	0	0.0 (0.0‐1.3)
No	—	—	—	—	226	98.7 (95.9‐99.7)	287	100 (98.7‐100)
Miscarriage								
Yes	—	—	—	—	<4	—	0	0.0 (0.0‐1.3)
No	—	—	—	—	227	99.1 (96.5‐99.8)	287	100 (98.7‐100)
Cesarean section^d^								
Elective	18	5.6 (3.5‐8.9)	4	3.8 (1.2‐10.1)	25	11.0 (7.4‐16.0)	27	9.4 (6.4‐13.5)
Acute	40	12.5 (9.2‐16.7)	11	10.6 (5.7‐18.5)	26	11.5 (7.8‐16.5)	45	15.7 (11.8‐20.5)
Stillbirth	4	1.2 (0.4‐3.2)	2	1.9 (0.3‐7.3)	<4	—	0	a
Preterm birth^d^	54	16.9 (13.0‐21.5)	15	14.4 (8.6‐23.0)	16	7.0 (4.2‐11.4)	43	15.0 (11.2‐19.8)
Small for gestational age^d^ (SGA)	43	13.4 (10.0‐17.8)	10	9.6 (5.0‐17.4)	15	6.6 (3.9‐10.9)	23	8.0 (5.3‐11.9)
Apgar score^d^ < 7 at 1 min								
Yes	28	8.8 (6.0‐12.5)	3	2.9 (0.7‐8.8)	13	5.9 (3.3‐10.1)	33	11.5 (8.2‐15.9)
No	292	91.3 (87.5‐94.0)	101	97.1 (91.2‐99.3)	207	90.4 (85.6‐93.7)	254	88.5 (84.1‐91.8)
Apgar score^d^ < 7 at 5 min								
Yes	7	2.2 (1.0‐4.7)	2	1.9 (0.3‐7.5)	4	1.8 (0.6‐4.9)	19	6.6 (4.1‐10.3)
No	313	97.8 (95.3‐99.0)	102	98.1 (92.5‐99.7)	217	98.2 (95.1‐99.4)	268	93.4 (89.7‐95.9)
Neonatal abstinence syndrome (NAS)^d^								
Yes	—	—	—	—	120	54.1 (47.3‐60.7)	287	a
No	—	—	—	—	102	45.9 (39.3‐52.7)	0	a

Abbreviations: CI, confidence interval; SD, standard deviation.

– data were not available for the Czech republic sample or there were less than four individuals in the Norwegian sample

a Given selection of comparison group in Norwegian sample not relevant to calculate

b Newborns born by women hospitalized with an ICD‐10 F11 diagnosis (opioid related disorders) as primary or secondary diagnosis during pregnancy who were not in OMT during pregnancy

c Newborns born with neonatal abstinence syndrome (NAS) by women who were not in OMT during pregnancy

d Live births

e Gestational age ≥ 37 weeks

### 3.3 Neonatal outcomes – regression analyses

Table 4 shows the results of linear and logistic regression analyses on birth outcomes comparing the OMT group to the comparison group in each country. The regression analysis of the OMT group versus the comparison group showed statistically significant effects in Norway: gestational age (unadjusted *b* = 0.93 weeks), preterm birth (unadjusted OR = 0.44), and low Apgar score at 5 minutes (unadjusted OR = 0.26). After adjustment for confounding factors the risk estimates of OMT exposure compared to no exposure were still significant; gestational age was nearly one week longer in OMT‐exposed (adjusted *b* = 0.96 weeks, 95% CI = 0.39‐1.53). OMT exposure also reduced the risk of preterm birth preterm birth (adjusted OR = 0.35, CI = 0.16‐0.75) and the risk of low Apgar score at 5 minutes (adjusted OR = 0.21, CI = 0.06‐0.78). OMT was not significantly associated with a difference in any of the neonatal outcomes in the Czech cohort.

**Table 4:** Linear^a^ and binary logistic regression^b^ comparing opioid maintenance treatment (OMT) to no such treatment during pregnancy in women with indications of opioid dependence in the Czech Republic (2000‐2014) and Norway (2004‐2013). Singleton pregnancies

	Czech republic		Norway	
	OMT vs No OMT (ref.)		OMT vs No OMT (ref.)	
	*b* a	95% CI	*b* a	95% CI
Gestational age^d^				
Unadjusted	0.03	0.60 to 0.55	**0.93 (0.94)** f	**0.47‐1.39**
Adjusted^c^	0.02	0.66 to 0.62	**0.96**	**0.39‐1.53**
Birth weight^e^				
Unadjusted	24.6	133.9 to 84.7	18.6	75.6 to 112.7
Adjusted^c^	26.6	149.1 to 96.0	62.2	50.8 to 175.1
Birth length^e^				
Unadjusted	0.02	0.56 to 0.60	0.05	0.40 to 0.50
Adjusted^c^	0.04	0.61 to 0.68	0.18	0.33 to 0.70
Head circumference^e^				
Unadjusted	0.30	0.16 to 0.74	0.13	0.41 to 0.15
Adjusted^c^	0.23	0.24 to 0.70	0.06	0.41 to 0.30
	**OR** b	**95% CI**	**OR** b	**95% CI**
Preterm birth^d^				
Unadjusted	1.21	0.65‐2.24	**0.44 (0.38)** f	**0.24‐0.81**
Adjusted^c^	1.25	0.63‐2.46	**0.35**	**0.16‐0.75**
Small for gestational age (SGA)^d^				
Unadjusted	1.46	0.71‐3.02	0.83	0.42‐1.63
Adjusted^c^	1.43	0.64‐3.18	0.58	0.26‐1.33
Apgar score^d^ < 7 at 5 min				
Unadjusted	1.14	0.23‐5.58	**0.26 (0.21)** f	**0.09‐0.76**
Adjusted^c^	0.92	0.16‐5.47	**0.21**	**0.06‐0.78**

significant findings are shown in bold

a *b* (regression coefficients) from linear regression

b Odds ratio (ORs) from binary logistic regression

c Adjusted for age, marital status, education, smoking

d Live births

e Gestational age ≥ 37 weeks

f The *b* or OR from crude regression analysis when restricted to the same study sample as in adjusted analysis

### 3.4 Supplementary tables

Tables S1 and S2 show background characteristics and the neonatal outcomes of the groups of women who were in OMT during pregnancy or who were in OMT before and after but not during pregnancy, and all pregnant women and their children (the general population of pregnant women) in both countries, respectively. The groups of women in both countries who at any time had received OMT differed from the pregnant women in the general population in that they had lower education, fewer were married or living with a partner, and more of them smoked (Table S1). Concerning neonatal outcomes in the Czech Republic, gestational age and growth parameters were quite similar in newborns of women who at some time had been exposed to OMT irrespective of if this was during pregnancy or not (Table S2). In Norway, the number of pregnancies where the mother had used OMT before or after pregnancy, but not during pregnancy, was very less. In both countries, the growth parameters were lower in OMT‐exposed newborns compared to newborns in the general population (Table S2).

## 4 DISCUSSION

In this study, we aimed to increase knowledge about the safety of OMT during pregnancy by examining neonatal outcomes concerning use of OMT during pregnancy using relevant comparison groups in two nationwide cohorts of pregnant women. We found no significant differences in neonatal growth parameters (birth weight, length, and head circumference) between OMT–exposed newborns and newborns of drug‐using pregnant women not in OMT during pregnancy. These findings were consistent in both countries. In the Norwegian sample, gestational age was nearly one week longer, and the odds of preterm birth and low Apgar score were lower in the OMT–exposed group. In the Czech Republic, these differences were not observed.

Our results on growth parameters are in contrast to previous research,^10^ which suggest better neonatal outcomes in children of mothers in OMT compared to children of women with opioid use disorders not in OMT. There can be several explanations for these differences, such as the more recent study period in the present study, the European setting, the use of different comparison groups, and the registry data design of our study.

Performing studies on safety of prenatal OMT exposure in another setting than in the United States, where the majority of early research has been conducted, might reduce the role of unmeasured confounding. In Norway and the Czech Republic, socioeconomic differences in the population are smaller than in the United States, and health care services are freely available for everyone. Thus, while opioid–dependent women in these countries have low socioeconomic status, they do not live in pronounced poverty.

In this study, we tried to identify comparison groups with quite similar risk factor profiles as women in OMT. The comparison group in the Czech Republic did not differ from the OMT group concerning background characteristics. In Norway, the women in OMT smoked more and had lower education. If background characteristics play an important role in affecting neonatal outcomes, the similar characteristics of the OMT and the comparison groups in our study suggest that the selected comparison groups were suitable for studying adverse outcomes of OMT during pregnancy. However, it should be noted that the comparison groups were identified by different criteria. In the Czech Republic, women with at least one diagnosis indicating drug dependence during pregnancy constituted the comparison group, while women who gave birth to a newborn with NAS comprised the comparison group in Norway. This difference combined with different treatment settings 23 can increase the generalizability of the findings. Nevertheless, the difference between the comparisons groups might have resulted in heavier opioid users constituting the comparison group in Norway, explaining the more positive effects of OMT in Norway than in the Czech Republic.

There were differences in gestational age, preterm birth, and Apgar score between the OMT–exposed and the nonexposed group in Norway. Recent reviews report that gestational age should be considered as a continuum concerning the risk and severity of adverse outcomes.^27^, ^28^ Even though the mean gestational age was within the range of term pregnancies in both the OMT groups and the comparison groups, it was almost one week longer in the OMT group than in the comparison group in Norway – even after adjusting for smoking and other sociodemographic factors. In Norway, OMT was also associated with reduced odds of preterm birth. These differences were not observed in the Czech sample. As mentioned above, a possible explanation may be that the Norwegian comparison group consisted of heavier drug–using mothers than in the Czech Republic. Further, a larger proportion of the Norwegian comparison group was reported to be smoking during pregnancy than in the Czech comparison group, and it is known that smoking during pregnancy increases the risk of prematurity.^29^ OMT was also associated with reduced odds of low Apgar score only in Norway. This difference may also be explained by the differences in comparison groups in the two countries. In the Czech Republic, the comparison group could include women who were abstinent during the last stage of the pregnancy.^30^ In contrast, the Norwegian group consisted of women whose children were born with NAS, indicating that the women were using drugs, quite likely opioids, up until the end of the pregnancy. This might have resulted in newborns being under the influence of maternal opioid use shortly after delivery, and this might have influenced the Apgar score in a negative direction.

### 4.1 Methodological considerations

Using information from the nationwide registries reduces the risk of selection and recall bias. By using registry data, all pregnant women are identified and followed‐up unless they move out of the country. This reduces the problem of selection both due to inclusion and to loss to follow‐up that might be especially important for pregnant women with opioid use disorders who more often have a transient lifestyle.^12^ Another strength with this approach is that health registries identify more women in OMT than can feasibly be included in clinical samples. While the samples of pregnant women in OMT in our study are among the largest to date, even larger samples are needed to study rare outcomes such as stillbirths and miscarriages.

The Norwegian Prescription Database only includes information on prescription drugs dispensed to outpatients. If a pregnant woman received all her OMT drugs during pregnancy at hospital or institution, this would not have been registered in the prescription database, and we would not have identified the woman as in OMT. We assume that this is the situation for a limited number of women in OMT.^22^ Some critical information such as smoking is underreported in the registries. Furthermore, some data like for instance use of alcohol and illicit drugs are not collected in the Norwegian registries.

In Norway, we used women who gave birth to children with NAS as the comparison group. Although it is not possible to attribute the cause of NAS to opioid exposure alone, opioids are the most common cause of NAS.^31^ Moreover, even though the women in the Norwegian comparison group might not have used opioids, but other drugs, they were much more similar to the pregnant women in OMT than pregnant women in the general population were and therefore more suitable as a comparison group.

Neonatal growth parameters in the OMT–exposed newborns were similar both to the outcomes in newborns of drug–dependent women not in OMT during pregnancy and to the groups of women using OMT outside of pregnancy, but not during pregnancy. However, when compared to the general population, all the newborns of women who had any indications of drug abuse before, during or after pregnancy, seem to have worse neonatal outcomes – regardless of whether the woman received OMT during pregnancy or not. Taken together, this might suggest that it is not the OMT drugs themselves that are associated with worse neonatal outcomes, but other factors related to opioid use, such as comorbidity, socioeconomic, and lifestyle factors.

Some critics have questioned the rationale for public health care services offering opioid maintenance treatment (OMT) during pregnancy. Our findings did not suggest that OMT results in worse outcomes for the newborns compared to no treatment. Moreover, we observed some important positive neonatal outcomes from OMT versus no OMT in the Norwegian sample. Seen in conjunction with the beneficial effects for the pregnant woman such as improved prenatal care adherence and obstetrical outcomes,^2^, ^32^, ^33^, ^34^ our findings support the prescription of OMT drugs to pregnant women with opioid use disorders.

## DISCLOSURES

None of the authors has anything to disclose.
